# The Role of Fetal, Infant, and Childhood Nutrition in the Timing of Sexual Maturation

**DOI:** 10.3390/nu13020419

**Published:** 2021-01-28

**Authors:** Valeria Calcaterra, Hellas Cena, Corrado Regalbuto, Federica Vinci, Debora Porri, Elvira Verduci, Chiara Mameli, Gian Vincenzo Zuccotti

**Affiliations:** 1 Pediatric and Adolescent Unit, Department of Internal Medicine, University of Pavia, 27100 Pavia, Italy; 2 Pediatric Unit, “V. Buzzi” Children’s Hospital, 20154 Milan, Italy; elvira.verduci@unimi.it (E.V.); chiara.mameli@unimi.it (C.M.); gianvincenzo.zuccotti@unimi.it (G.V.Z.); 3 Laboratory of Dietetics and Clinical Nutrition, Department of Public Health, Experimental and Forensic Medicine, University of Pavia, 27100 Pavia, Italy; hellas.cena@unipv.it (H.C.); debora.porri01@universitadipavia.it (D.P.); 4 Clinical Nutrition and Dietetics Service, Unit of Internal Medicine and Endocrinology, ICS Maugeri IRCCS, 27100 Pavia, Italy; 5 Pediatric Unit, Fond, IRCCS Policlinico S. Matteo and University of Pavia, 27100 Pavia, Italy; corrado.regalbuto01@universitadipavia.it (C.R.); fede90vinci@gmail.com (F.V.); 6 Department of Health Sciences, University of Milano, 20142 Milano, Italy; 7 “L. Sacco” Department of Biomedical and Clinical Science, University of Milan, 20157 Milan, Italy

**Keywords:** fetal, neonatal, nutrition, diet, timing, puberty, sexual maturation

## Abstract

Puberty is a crucial developmental stage in the life span, necessary to achieve reproductive and somatic maturity. Timing of puberty is modulated by and responds to central neurotransmitters, hormones, and environmental factors leading to hypothalamic-pituitary-gonadal axis maturation. The connection between hormones and nutrition during critical periods of growth, like fetal life or infancy, is fundamental for metabolic adaptation response and pubertal development control and prediction. Since birth weight is an important indicator of growth estimation during fetal life, restricted prenatal growth, such as intrauterine growth restriction (IUGR) and small for gestational age (SGA), may impact endocrine system, affecting pubertal development. Successively, lactation along with early life optimal nutrition during infancy and childhood may be important in order to set up timing of sexual maturation and provide successful reproduction at a later time. Sexual maturation and healthy growth are also influenced by nutrition requirements and diet composition. Early nutritional surveillance and monitoring of pubertal development is recommended in all children, particularly in those at risk, such as the ones born SGA and/or IUGR, as well as in the case of sudden weight gain during infancy. Adequate macro and micronutrient intake is essential for healthy growth and sexual maturity.

## 1. Introduction

Puberty is a crucial developmental stage in the life span that leads to reproductive and somatic maturity. [1,2,3,4]. Timing of puberty is modulated by interplay and balance between hormones, central neurotransmitters, and environmental factors inducing the hypothalamic-pituitary-gonadal (HPG) axis maturation. [2]. These interactions take place at early developmental stages and impact on pubertal timing [4]. 

Endocrine systems programming arises during critical phases of fetal development and may therefore be influenced by abnormal intrauterine growth. Birth weight is a major indicator of growth estimation during fetal life. Intrauterine growth restriction (IUGR), and small for gestational age (SGA) are terms used to describe respectively a lower fetal growth than expected [1], and a neonate with a birth weight below two standard deviations (2SD) below the mean or below the 10th percentile of a population-specific birth weight for specific gestational age, which are common manifestations of small fetuses [1]. As reported by the literature, restricted prenatal growth may cause permanent endocrine axes alteration, which in turn affect pubertal development. IUGR and SGA conditions are sensitive to nutrition imbalances, in particular to maternal nutritional deficiencies during peri-implantation and placental development [5,6,7]. Successively, breastfeeding or formula feeding, along with early life healthy nutrition and nutritional status during infancy and childhood, impact on sexual maturation timing and successful reproduction at a later time [8].

This narrative review aimed at combining evidence of fetal and neonatal nutritional status on sexual maturation physiology and timing.

## 2. Methods

In order to obtain a wide perspective of the role of fetal and neonatal nutrition on sexual maturation timing, each author identified and reviewed the most appropriate published studies (original paper and reviews) in English language about timing of sexual maturation considering fetal and neonatal nutritional status and birthweight. The following keywords were used to search for papers published up to October 2020: birthweight, IUGR, SGA, nutrition, diet, puberty onset, puberty timing, pubertal development, hypothalamic-pituitary-gonadal axis, lactation, macronutrients, micronutrients, nutrient intake and requirements. Electronic databases PubMed, Scopus, EMBASE, and Web of Science were used for research. All inputs were collected and the drafted preliminary version was discussed among authors [6]. The final version was then recirculated and approved by all the co-authors.

## 3. Physiology of Puberty

Puberty is a transitional period in children resulting from the hypothalamic-pituitary-gonadal (HPG) axis activation [2,9].

The HPG axis in children is completely functional during fetal life. At birth, in the absence of placental steroids suppressing the HPG axis, there is a sudden activation of that axis that causes an increased production of steroidal hormones defined as minipuberty. This transient activation of HPG axis starts approximately one week postnatally and takes places during the first six months of life [2,9,10]. The increase of luteinizing hormone (LH) and follicle-stimulating hormone (FSH) provokes testosterone secretion by testis in boys and estradiol by ovaries in girls. Hormone concentrations during minipuberty influence genital organ development and fertility, somatic development, and body composition in the first 12 months of life, as well as cognitive development [2,9,10].

**Figure 1:**
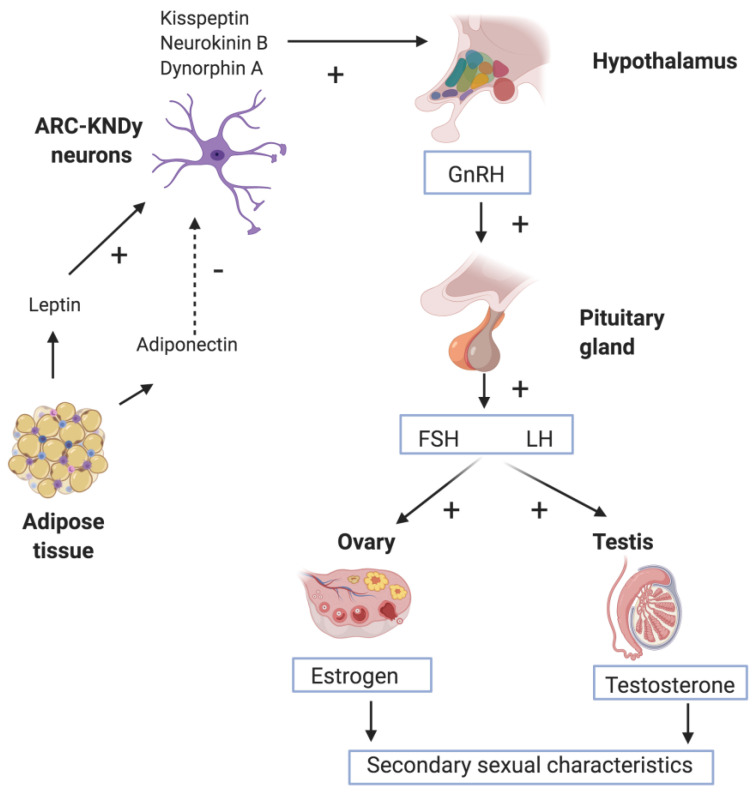
Stimulation of the hypothalamic-pituitary-gonadal (HPG) axis. The pulsatile release of hypothalamic gonadotropin-releasing hormone (GnRH) activates production and secretion of luteinizing hormone (LH) and follicle-stimulating hormone (FSH) by the pituitary gland, inducing gonadal production of estrogen and testosterone in girls. Onset of GnRH release is driven by a great number of different factors, including arcuate nucleus (ARC) kisspeptin/neurokinin B/dynorphin A (KNDy) neurons and adipokines. Created with BioRender.com.

HPG axis activation is silenced after few months. Later in infancy, HPG axis becomes inactive during the first five years of life, until its successive activation in adolescence. At this time, FSH and LH secretion from adenohypophysis is stimulated by gonadotropin-releasing hormone (GnRH) released from the hypothalamus in a pulsatile way in order to avoid downregulation of its receptor in the pituitary gland [2,9,10]. Evidence suggests arcuate nucleus (ARC) kisspeptin/neurokinin B/dynorphin A (KNDy) neurons as key players in GnRH pulse generation, as well as in pulsatile GnRH/gonadotropin secretion regulation, thus triggering pubertal in mammals including rodents, ruminants, and primates [7]. FSH and LH induce spermatogenesis and oogenesis along with testosterone and estradiol release, respectively, in males and females [8,9], Figure 1.

According to Marshall and Tanner, the five the stages of puberty include somatic changes in breast, pubic hair, and genital development, both in girls and boys. Puberty occurs at average 11.15 (±1.10) years in girls and in boys at 11.64 (±1.07) years [11]. Although some physiological variation is known, puberty development usually endures 3–4 years and comprises a sequence of events that proceed in a typical foreseeable order [10]. The first pubertal sign is generally testicular enlargement in boys and thelarche (breast development) in girls. Menarche is considered the final marker of puberty in females, while in males, enlargement of testis is followed by growth velocity escalation and consequent spermarche, voice changing, and facial hair growth [2]. The development of pubic and axillary hair is also incorporated in the Tanner classification, but it is not a recommended marker of puberty onset. In both sexes, pubarche is determined by adrenal androgen production increase [2].

The normal timing of puberty is crucial in psychological and physical development. Children with early puberty are at greater risk for psychiatric problems (such as depression, anxiety, and bulimia [12]), social isolation, early sexual behavior, and are at risk of potential abuse by adults [13,14]. Additionally, in both sexes, short adult stature, due to premature epiphyseal closure, is another possible consequences of precocious puberty [15].

The secretion of GnRH is driven by different inter-related and complex stimuli, including leptin, kisspeptin, neurokinin B, and glutamate, along with numerous glial signaling molecules, Figure 1. These enhanced processes are coupled with the loss of other inhibitory signals within the arcuate nucleus, resulting into a feedback directed to GnRH pulse generator [16].

Two metabolic hormones, called leptin and kisspeptin, seem to be closely associated to early onset of puberty and premature nutritional insults. The actions of these hormones are determined by different genetic controls, which eventually depend on numerous transcriptional regulatory configurations, similar to other arrangements found in other gene networks [17]. These arrangements are affected by early environmental factors, programming particular phenotypical expression. This persistent programming pattern is named the ‘epigenome’ [18]. This epigenetic configuration is one of the mechanisms that might affect leptin and neuropeptide kisspeptin action.

Kisspeptin action and its G-protein coupled receptor 54 (GPR54), are connected to the proper timing of puberty onset. The kisspeptin/GPR54 system, has a central role in the regulation of GnRH secretion in humans as well as in other mammals.

Several studies have shown that the central or systemic administration of kisspeptin increases GnRH and gonadotropin secretion in prepubertal and adult animals [19,20,21,22,23,24].

The kisspeptin system is influenced by premature malnutrition, both over and undernutrition. A study demonstrated also that fetal undernutrition interferes with the production of kisspeptin, directly affecting timing of puberty onset in mice [25]. Besides, pubertal timing is normalized by chronic central injection of kisspeptin [26].

Concerning leptin levels, it has been recently reported that concentrations of soluble leptin receptor may be responsible for the critical signals in puberty timing [27]. Leptin concentration is correlated with birth weight and increases markedly just before puberty onset [28]. Leptin is affected by early life nutritional nods and it is considered a crucial factor for both puberty onset and energy balance, besides being a key player in fertility [28,29].

Recently, it has been reported that leptin can also be considered a positive regulator of the kisspeptin/GPR54 system [9]. The link between leptin levels prior to puberty onset and the specific actions of kisspeptin, as well as the other coordinated chemical cascade anticipating puberty onset, remains not fully elucidated [30]. Moreover, the system that underpins puberty timing is complex and structured. Good maternal and early life nutritional status likely regulates at least two central regulatory chemicals [31].

## 4. Fetal Growth and Timing of Puberty

Altered fetal nutrition and endocrine system lead to developmental modifications that everlastingly affect structure, physiology, and metabolism. Interplay between hormones and nutrition during critical periods of growth, like fetal life or infancy, is fundamental for what concerns metabolic adaptation response control and pubertal development expectation [32].

There is increasing evidence suggesting that the prenatal and early postnatal period are a perfect interval, receptive to long-term ‘programming’ of pubertal development [33]. During pregnancy, maternal nutrition also impacts fetal growth, both directly and indirectly, respectively by providing nutrients to the embryo and by regulating endocrine mechanisms expression affecting fetal absorption and exploitation of nutrients [1].

Several reports have shown that prenatal exposure to adverse environmental factors during pregnancy, like factors responsible for children born SGA and/or IUGR or detrimental factors during lactation, affect puberty timing [1,2,3].

### 4.1. Small for Gestational Age and Pubertal Timing

SGA is a term used to define a neonate with birth weight lower than the 10th percentile for that gestational age or 2 SD below the population norms on the growth charts [2]. SGA infants do not necessarily have growth restriction in utero; in fact, they may be constitutionally small [2]. The definition of SGA includes individuals with a low plotting birth weight but normal plotting birth length or, contrariwise, neonates who may have been born with a normal birth weight but short in length (<2.5 SD below the mean) [34]. Indeed, some SGA children are both short in length and low in weight. Consequently, infants born SGA may be either defined SGA with a low birth weight, SGA with a low birth length, or SGA with a low birth weight and length. As for weight at birth, low birth weight (LBW) is used for newborns who weigh less than 2500 g at delivery, independently of gestational age at birth. Other related terms comprise very low birth weight (VLBW, less than 1500 g) and extremely low birth weight (ELBW, less than 1000 g) [35]. Normal birth weight at term delivery is about 2500–4200 g [35]. Noteworthy SGA is not to be considered a synonym of LBW, VLBW, or ELBW, however almost one-third of LBW infants, who weigh less than 2500 g, are also SGA [35,36].

Being born SGA may predispose a child to a series of puberty alterations, such as precocious adrenarche and puberty, and earlier onset of menarche [37,38,39,40,41,42,43,44].

However, SGA children are more susceptible to show precocious pubarche and earlier pubertal development onset, as well as menarche or faster puberty progression [37,38,39,40,41,42,43,44].

In scientific literature, association between fetal growth and pubertal development has been evaluated mainly investigating females, especially considering age of menarche in girls [37,38,39,40,41,42,43,44], highlighting that children born with low birth weight or SGA have been associated with earlier age at the onset of menarche. Pubertal onset and age at menarche frequently are advanced by about 5–10 months in girls born SGA, particularly if there has been an important catch-up growth for nonadjusted age during the first months of life [45]. Most preterm infants show catch-up growth, defined as reaching a standard deviation score higher than −2 z-score than the reference population, within the first two years of life [46]. Hvidt JJ et al. [47], in a study conducted on 15822 patients, deduced that the girls born small for gestational age (SGA) reached pubertal markers at an earlier mean age than girls born appropriate for gestational age (AGA), except for breast development, while boys born SGA and large for gestational age (LGA) achieved puberty prior than boys born AGA. The steps and timing of pubertal events looked similar to the ones of girls born AGA [47]. 

Persson et al. [48] also showed that girls with a low weight at birth or born short for gestational age started puberty earlier compared to normal girls, although statistical difference was reached only in girls born light for gestational age. In a longitudinal analysis conducted by Lazar et al. [49] on a group of 76 SGA and 52 AGA children, from early childhood to the completion of puberty, most recruited children in both groups entered puberty at a normal age, the duration of puberty was similar, but some of the subjects in the SGA group begun puberty significantly earlier than AGA children, with a significant difference in both genders. Moreover final height in the SGA group was compromised compared with their target height. Bhargava et al. [50] showed that younger age of puberty onset was associated to higher birth weight z-score for gestational age. Indeed subjects with lower birth weight showed quicker linear growth, and earlier pubertal development [50]. Other prepubertal factors, including gestational age, BMI, and bone age delay during childhood were not correlated with age of puberty onset [51]. In a report study based on postnatal growth of 3,650 healthy Swedish children, 87% of those born SGA, showed full catch-up growth within the first 24 months of life, achieving puberty at a normal or younger age and reaching mean final height of −0.7 SDS. The subgroup of those born SGA (13%), remained below −2 SDS throughout childhood and reached puberty somewhat early [52,53]. Koziel S et al. showed that earlier age at menarche was observed in girls with lower birth weight and higher BMI at 14 years of age, compared to AGA girls or girls born SGA with a lower current BMI [37].

Sloboda DM et al., described the impact of BMI on menarche timing in SGA girls—in those with lower birth weight and higher BMI, menarche occurred earlier [39]. In the study conducted by Ibanez et al. on girls with early pubarche, menarche before age 12 was three-fold more prevalent among those born SGA; their age at menarche was 8–10 months more advanced than girls of normal birth weight [54]. Puberty starts within normal age range in SGA children, but its onset occurs generally earlier than AGA children.

It has been proposed that quick weight gain in childhood could lead to precocious pubarche in predisposed individuals. Nevilleet al. reported that being born SGA based on weight and/or length is an independent risk factor for early puberty [55]. Infants presenting rapid postnatal weight gain have, so far, shown the highest adrenal androgen concentration [56,57,58,59]. Ong et al. [60] have shown a correlation between lower birth weight and higher adrenal androgen concentration in both genders. Many authors agree that there is a relationship between premature pubarche, precocious adrenarche, and being born SGA. This association could be due to increased central adiposity, increased insulin resistance, and increased IGF-I levels stimulating adrenal androgen production and early pubic development.

Early accumulation of visceral fat following postnatal catch-up growth in SGA children, leading to insulin resistance and hyperinsulinism, could be largely responsible for hyperandrogenic state development in SGA girls [61].

Regarding SGA infants both with or without catch-up growth, lbersson-Wikland K a et al. reported that children who remained short throughout childhood presented a relatively early age puberty onset both with or without catch-up growth [52]. On the other hand, a longitudinal analysis comparing pubertal course of constantly short children born SGA, compared to children born AGA with idiopathic short stature, recurrently monitored from early childhood to puberty achievement, showed that puberty was reached at normal age for most children in both groups, with no differences in duration. [49].

Concerning boys, data are too scarce to conclude on the way or extent of the association between timing of puberty and fetal growth [62,63]. Boys born SGA are at risk for high FSH and low inhibin B levels; timing of puberty is described normal but testicular volume and adult height are often beneath target [64]. Relationship between low birth weight, testicular dysgenesis syndrome, cryptorchidism, hypospadias, testicular cancer in adult life [65], and subfertility has been reported [62].

Thus, early surveillance and monitoring of the pubertal stages are highly recommended in SGA children.

### 4.2. IUGR Born SGA or AGA and Timing of Puberty

IUGR is usually defined as a retardation of fetal growth and development and/or its organs during gestation, as documented by at least two intrauterine growth measurements [66]. It is therefore a clinical condition in which an unfavorable intrauterine environment may somehow influence fetal development that fails to reach its genetic potential growth, with possible repercussions on future health status.

It is important to emphasize that not all IUGR children are born SGA and not all children born SGA are IUGR. However, in most studies, these terms have been used interchangeably, leading to difficult interpretation of the results.

IUGR children, particularly if they show a catch-up growth in early life, develop higher risk for long term complications including short stature, metabolic syndrome, insulin resistance, diabetes type 2, and cardiovascular diseases [67]. There is increasing evidence that exposure to endocrine chemical disruptors (ECD) may be responsible for IUGR, and therefore may be related to late health effects, such as changes in puberty development [68]. Literature studies have shown that there is a correlation between IUGR, insulin resistance and compensatory hyperinsulinism that occurs late due to rapid weight gain in early childhood, which would be responsible for increasing the bioavailability of sex hormones and, finally, for acceleration of puberty [69,70].

Some experimental studies showed that poor fetal nutrition permanently affects growth and development of ovarian follicles. It is hypothesized that fetal growth delay in the rat IUGR model occurs in a delicate period and is related to germ cell increase, which can therefore permanently alter the final number of follicles. Current studies assume that these results in the female rat IUGR are related to malnutrition that occurs mainly during the second trimester in human beings [49,52]. The consequent reduction in follicles number may be responsible of early ovarian failure patterns [71,72,73]. In addition, ARC, situated in the mediobasal portion of hypothalamus, is sensitive to perinatal nutritional status affecting Kisspeptin 1 hypothalamic expression and puberty timing in female rats [74]. Undernutrition during intrauterine life negatively affects the production of kisspeptin in rats and thus indirectly impacts timing of puberty [16]. On the other hand, in a study performed on IUGR lambs, no difference was found in early puberty and ovarian function in females, while in males testosterone values and testicular mass were lower than controls [75].

However, a prospective human study, conducted by Hart R et al., who recruited 230 girls and studied their ovarian reserve, ovarian ultrasound data, and levels of anti-Mullerian hormone (AMH), FSH, and inhibin B, showed that these parameters are not affected by changes in intrauterine growth [76].

Ibanez L et al. [77] showed that nonobese girls with an early puberty pattern or a history of precocious pubarche, presented a low weight at birth, therefore precocious pubarche, hyperandrogenism and insulin resistance could be considered consecutive manifestations of IUGR severity.

In other studies, it has been reported that IUGR girls showed clinical patterns of ovarian hyperandrogenemia and polycystic ovary syndrome (PCOS) phenotype, while IUGR males more often showed infertility [78,79]. Some studies also displayed that IUGR status was apparently associated with high levels of FSH in early childhood in boys and girls born SGA [80]. In fact, FSH secretion at three and six months in both males and females IUGR was two–three times higher than AGA controls [80]. A study conducted by Linda S. Adair, on the basis of data collected during Cebu team study [38], showed early menarche presentation in IUGR girls, especially in those with accelerated postnatal growth. The significant correlation between the measurements at birth and menarche, despite other influencing factors, gives more credit to the hypothesis that growth curves and pubertal maturation are always influenced by intrauterine environment [79]. Changes during puberty in IUGR births disclose correlation with chronic diseases that occur in adulthood such as diabetes type 2 or coronary heart disease, as well other ones, including polycystic ovary syndrome (PCOS) and short stature [80]. Puberty is a delicate transition phase, still sensitive to the problems that occurred during intrauterine life. IUGR children have higher risk of developing early puberty (before the age eight in girls and nine in boys), precocious pubarche, polycystic ovary syndrome that will affect fertility in girls, and small testicular size with possible male infertility. Therefore, IUGR children and adolescents should undergo close follow-up to monitor the development of negative consequences, including pubertal problems and abnormalities of reproductive function [80].

## 5. Neonatal and Early Infancy Nutrition and Puberty

Breast milk is ideally the first food that newborns consume, compatibly with maternal condition and personal choices, and it is acknowledged to be the best source of energy and nutrients through the first year of life, providing benefits on growth, immunity, and neurodevelopment [81,82]. Human breast milk supplies the baby with immune cells, stem cells, skin cells, and bacteria that have both short- and long-term effects on health [83,84]. The Word Health Organization (WHO) recommend exclusive breastfeeding from birth to six months of life and complementary breastfeeding until two years [85]. Two European prospective cohort studies aimed at highlighting whether breastfeeding also affects early puberty onset [57,86] found no independent relationship between breastfeeding and menarche age, nor did Man Ki Kwok and colleagues [87] outside Europe, examining whether breastfeeding was associated with pubertal onset in the Hong Kong’s Children 1997 cohort [88].

Despite these findings, breastfeeding has recently been referred to as a critical independent factor in postponing sexual maturation in girls at higher risk of early puberty onset [89]. Authors observed an association between breastfeeding and thelarche at later age in 3331 mother–daughter pairs, after adjusting for maternal age at delivery, education level, and ethnicity, revealing that girls who were not breastfed were more likely to experience earlier thelarche than girls who were breastfed for six months [89]. In another study on 219 Korean children [10], authors found that subjects who were breastfed for over six months had lower odds of Tanner stage attainment ≥2 at nine years, compared with those who stopped breastfeeding before six months. Furthermore, results from the Cebu Longitudinal Health and Nutrition Survey showed a 6% risk decrease of premature menarche with each one-month increase in breastfeeding duration (hazard ratio = 0.94; 95% CI 0.90, 0.98) [90]. Therefore, we can postulate that evidence supports the protective role of breastfeeding against early puberty onset.

Overfeeding in infancy can result in an early start of puberty in both sexes [91]. Evidence has demonstrated that children born with low birth weight, who were overfed with rapid infantile catch-up growth, showed increased body fat and leptin resistance during childhood influencing timing of puberty [92,93,94] In contrast, children without appropriate nutrient provision, with consequent delayed catch up growth, displayed body weight, body fat, and plasma leptin within the normal range [27].

Emmett PM et al. [95], showed that rapid weight gain during infancy was linked to raised risk of obesity at five and eight years, along with increased insulin resistance, overstated adrenarche, and decreased sex hormone-binding globulin levels. Physiologically, increased levels of IGF-I and adrenal androgen possibly enhance the aromatase activity and free sex steroid levels, stimulating GnRH release [95,96]. As reported by Zheng [97], the significant associations between rapid weight gain and obesity remained after adjusting for child birth weight, with consequent influence on puberty timing.

All this might be explained and linked to the well documented actions of breastfeeding in reducing risk of childhood obesity [98], which itself promotes early sexual maturation.

According to this hypothesis, timing of introduction and type/order of complementary food should be considered, since they have been associated with increased risk of obesity in pediatric age [99], mostly by shaping gut microbiota colonization and altering gut microbial function and interactions of the microbiome with the host metabolic functions [100,101]. Consequently, it is possible to hypothesize a relationship between early introduction of solid foods (before four months of life) and risk of precocious puberty, although more research is needed to confirm this hypothesis.

## 6. Nutrient Intake during Childhood and Timing of Puberty

### 6.1. Macronutrient Intake

During infancy and childhood, sexual maturation is sensitive to feeding, requiring adequate nutrients intake for healthy growth.

A recent metanalysis systematically investigated the relationships between nutrient intake and childhood early menarche onset, revealing a significant association with high energy intake, animal protein, and iron [102]. In particular, protein intake needs to be considered, since protein hypothesis establishes that early protein intake inclines children to adiposity rebound before the onset of pubertal marker [103,104]. Considering the type of proteins, animal sources, including meat and dairy products, with high-quality proteins, have been investigated in relation to sexual maturation. Wiley et al. [105], using data of the National Health and Nutrition Examination Survey on 2057 girls from the United States, found that higher milk consumption at 5–12 years was associated with an earlier age at menarche.

Similarly, two other studies [106,107], with a smaller sample size, reported that protein intake from dairy products was associated to earlier pubertal growth. As for meat intake, according to the Avon Longitudinal Study of Parents and Children [108] involving 3298 girls, results showed that those with the highest intake of meat at age three and seven years showed greater odds of menarche at age 12.5 years compared to the counterparts consuming less meat. On the other hand, Carwile and colleagues did not find any association between peripubertal meat intake and menarche age in a big sample of 5583 American girls [109].

However, appropriate consumption of animal-based food sources of proteins, including dairy products, have shown to ensure growth and development in children [110]. In particular, dairy products, including milk, are important suppliers of many key nutrients, some of which are particularly important at certain stages of life. Moreover, low consumption of dairy products may contribute to suboptimal intake of calcium, magnesium, iodine, and other essential nutrients [111].

Several studies have reported that large consumption of sugar and sweet beverages among childhood worldwide exert negative health effects, [112,113,114,115,116,117,118,119,120,121,122,123,124,125,126,127] but the effects on puberty onset are still controversial. Only one prospective study found an association between early sexual development and consumption of sugar-sweetened beverages in a sample of 5583 American girls. Subjects consuming >1.5 servings sugar-sweetened beverages daily had an estimated earlier menarche of about 2.7 months, compared to those consuming ≤2 servings sugar-sweetened beverages weekly [118].

Even when considering lipid intake and its contribution to early pubertal development, results are conflicting. Some authors reported that total lipid intake in childhood influences early menarche onset [119,120], however other findings have shown no association at all [121,122]. Furthermore, a recent study, aimed at evaluating the influence of diet in childhood and the development of prostate cancer, found an association between a diet rich in lipids (32–36%) and animal proteins (9–10%) and early onset of puberty [123].

It is worth considering that girls with energy malnutrition seem to experience menarche at a later age than well-fed girls [124,125].

### 6.2. Micronutrient Intake

Prepubertal micronutrient status may influence sexual maturation timing, while micronutrients requirements increase during puberty. Evidence disclosed that iron, zinc, and calcium are essential micronutrients for growth and sexual maturity, and their requirements rise drastically during the growth spurt [27].

The above-mentioned systematic review [126], also showed that higher iron intake was associated with early menarche onset. Considering blood ferritin as an adequate index of iron storage in the absence of inflammation [127], a longitudinal study conducted on 3202 girls from the Bogotá School Children Cohort (BoSCCo) found an association between high ferritin and later menarche, reflecting a relationship between nutritional status and not only nutrient intake, but also timing of puberty. It is well known that animal foods are the main sources of some nutrients involved in early puberty process, including iron and proteins. However those same foods are also sources of vitamin B12 [128], which has shown no association with age at menarche [102].

The role of vitamin D in pubertal development has been also investigated. It is well known that there are Vitamin D receptors in ovary, uterus, placenta, testis, and pituitary glands, and for this reason they are acknowledged to play a key role in reproduction [129,130]. However, strong scientific evidence supporting a significant role in precocious puberty for either vitamin D or vitamin A does not exist in the literature [131]. Studies available are few and dated, and further well-conducted studies with a primary aim of investigating micronutrients role, as well as phytochemical compounds with well-known antioxidant actions, in sexual development are needed, especially because consumption of fruit and vegetables in childhood remains below recommendations in most countries, although their beneficial effects on health are widely demonstrated. Nevertheless assessing fruit and vegetables consumption, interesting results have been found with regard to fiber intake, which is known to modulate estrogen circulating levels, which in turn influence puberty onset mediated by HPG system [131]. Authors of the above mentioned systematic review [132], however, did not find any statistical significant difference of the dose-response effect of fiber intake in childhood and delayed menarche onset.

## 7. Conclusions

Puberty timing may be set during fetal life or early infancy and it can be altered by changes in nutrition, body composition, and size in childhood. Early nutritional surveillance and monitoring of pubertal development is recommended in all children, particularly in at-risk ones, such as children born SGA and/or IUGR, and when a rapid weight gain in infancy occurs. Appropriate micro- and macronutrients intake is also essential for growth and sexual maturity. A balanced diet is critical and nutritional excesses/deficiencies or unhealthy dietary patterns may impact on puberty, delaying growth and sexual development, affecting also immune function and causing significant morbidities later on. Future studies should focus on nutritional assessment and effective satisfaction of requirements to fulfill ‘optimal functions’ and prevent chronic adult diseases.

## Data Availability

Not applicable.
